# Treatment of canine pancreatitis using membrane-free stem cell extract and its anti-inflammatory effect

**DOI:** 10.3389/fvets.2025.1590703

**Published:** 2025-06-02

**Authors:** Yongsoo Choi, Miae Lee, BoMyoung Kim, Youngsil Kim

**Affiliations:** ^1^Yong Animal CTMRI Medical Center, Changwon, Republic of Korea; ^2^The Prime Animal Hospital, Busan, Republic of Korea; ^3^T-Stem Co., Ltd., Changwon, Republic of Korea

**Keywords:** canine pancreatitis, acute pancreatitis, canine-specific pancreatic lipase (cPL), C-reactive protein (CRP), stem cell, membrane-free stem cell extract (MF-STEM)

## Abstract

Canine pancreatitis can lead to the digestion of nearby organs, and if untreated, it can develop into peritonitis. Widely used treatment as of now for acute pancreatitis (AP), however, largely depends on supportive treatment such as fluid therapy, antibiotics, and sufficient nutrient intake. Membrane-free stem cell extract (MF-STEM) is a protein mixture and other bioactive molecules derived from human adipose stem cells. MF-STEM is known for its anti-inflammatory and regenerative effects. This study was a vehicle-controlled clinical study. One hundred and forty-five canine pancreatitis patients with pancreatitis participated in this study with owners’ consent. Following the owners’ consent, 63 dogs received MF-STEM cell therapy with conventional therapy, and 82 dogs received conventional therapy. All participants had their cPL and CRP measured to evaluate their clinical progression. At the time of discharge, the survival rate in the MF-STEM cell therapy group survived (survival 92% and mortality 8%) more than the control group (survival 46% and mortality 54%). The authors suppose that MF-STEM cell increases survival of pancreatitis patients by modulating inflammatory cytokines. There were no side effects observed, such as allergic reactions and hypersensitivity reactions.

## 1 Introduction

Canine pancreas is a friable organ. It is vulnerable to high-fat diet, ingestion of foreign objects, drugs, and toxins. Furthermore, concurrent diseases such as hyperadrenocorticism, hypothyroidism, and diabetes mellitus worsen its condition. Inflammation of the pancreas leads to the premature activation and release of digestive enzymes. These enzymes cause local damage to the exocrine pancreas, resulting in pancreatic edema, bleeding, inflammation, necrosis, and surrounding fat necrosis. The ensuing inflammatory process leads to the recruitment of white blood cells and cytokine production. Initial symptoms include abdominal pain, anorexia, vomiting, diarrhea, and melena. The current treatment for acute pancreatitis mainly provides supportive care and prevention of its complications by administering fluids, antiemetics, gastrointestinal protectants, and antibiotics. However, if the patient fails to cure itself, pancreatitis can progress to systemic inflammatory response syndrome (SIRS), leading to peritonitis. The SIRS refers to an inflammatory response throughout the body caused by various causes such as pancreatitis, systemic trauma, burns, and severe infections. When infection is combined with SIRS, it is called sepsis. Sepsis is a life-threatening medical emergency. In severe acute pancreatitis cases, sepsis can develop and result in death (1). The known mortality rate in the case of acute pancreatitis is between 27 and 66.6% (2, 3).

Histopathology of the pancreas is the gold standard for diagnosing pancreatitis. However, the owners are reluctant to agree due to its cost and the anesthetic risk. Thus, pancreas tissue is sampled when it is difficult to distinguish between pancreatitis and pancreatic cancer (4). Commonly, the diagnosis of AP involves patient history (e.g., gastrointestinal signs such as vomiting and diarrhea), clinical presentation (e.g., acute abdominal pain, dehydration, and borborygmi), blood tests (including CBC, serum chemistry, and venous blood gas analysis), abdominal radiography, and abdominal ultrasound. Canine-specific lipase (cPL) can specifically evaluate pancreatitis (5), although abdominal ultrasound should also be performed to assess the pancreas and its surrounding organs. While various options are available, Vcheck cPL can provide a quick result and quantify the amount of lipase (6). C-reactive protein (CRP) is another important blood marker for assessing the patient’s status. Measuring CRP allows clinicians to evaluate whether the pancreatitis patient has developed peritonitis as a complication and determine the severity, and assess the clinical progression of the disease (7).

Compared to other types of stem cells, human adipose stem cells (HASCs) are easier to collect and have greater capacity, making them more accessible. They also exhibit excellent anti-inflammatory and regenerative effects, which have attracted significant attention in clinical settings (8). In a previous study, component analysis of MF-STEM was performed to identify the total protein profile. MF-STEM contains nine proteins associated with anti-inflammatory effects and 36 proteins associated with regenerative (wound healing) effects (9). Furthermore, other recent studies have reported the anti-allergic (10), anti-inflammatory, immunomodulatory, angiogenic, and wound healing effects (11) of HASCs.

Current stem cell technology generally includes first-generation approaches, which use autologous adipose-derived stem cells directly, and the second-generation technology, which involves culturing, proliferating, and administering them to the patient. However, since antigens that cause immune rejection are attached to the cell membrane, they can generally only be used by the originating patient. To overcome immune rejection and develop a drug that can be widely used in people and animals, a technology to remove the cell membrane of stem cells has been developed. Membrane-free stem cells are protein components within stem cells. After breaking the cell membrane, unnecessary components that cause immune rejection are removed using a microfilter to purify only the effective ingredients. The purified effective ingredients are then freeze-dried and made into a powder form that is easy to store and distribute. This is the third-generation stem cell, membrane-free stem cell extract (MF-STEM) (9, 12–14).

The MF-STEM used in this study was manufactured and provided by T-Stem Co., Ltd. (Changwon, South Korea). Non-clinical and clinical trials for MF-STEM were completed in 2020, and it received approval for use in dogs from the Korea Quarantine Agency in 2021, receiving manufacturing and product approval for veterinary medicine. Its safety was confirmed through 22 large-scale toxicity and safety tests conducted by a Good Laboratory Practice (GLP) certified institution (15). It is currently sold under the trade name ‘T-STEM PET’. The MF-STEM cell has been proven to have a therapeutic effect on canine atopic dermatitis (12) and osteoarthritis (16).

Since the current treatment protocol for canine acute pancreatitis focuses on supportive care, recovery is challenging, and it often progresses to systemic inflammatory response syndrome (SIRS), leading to sepsis and death. Therefore, we hypothesize that the administration of the MF-STEM cell will regulate the inflammatory response and help the canine pancreatitis patients to recover. This study aims to investigate the therapeutic effects of MF-STEM on canine pancreatitis patients by comparing cPL, CRP values, and survival of the control and test group.

## 2 Methods

### 2.1 Experimental design

This study is a multicenter clinical study using MF-STEM. The experimental subjects were dogs diagnosed with pancreatitis who visited Yong Animal CTMRI Medical Center and The Prime Animal Hospital from January 2021 to December 2024. Among them, only the in-patients were considered as the study participants. A total of 145 dogs participated, including 63 in the test group and 82 in the control group. The study participants were allocated in each group following the owners’ consent. All owners signed a consent form for this clinical study before participating. The animal study was reviewed and approved by the Clinical Trial Ethics Committee of the Korea Stem Cell Industry Association (Approval number: KSIA-202012-645).

**Figure 1:**
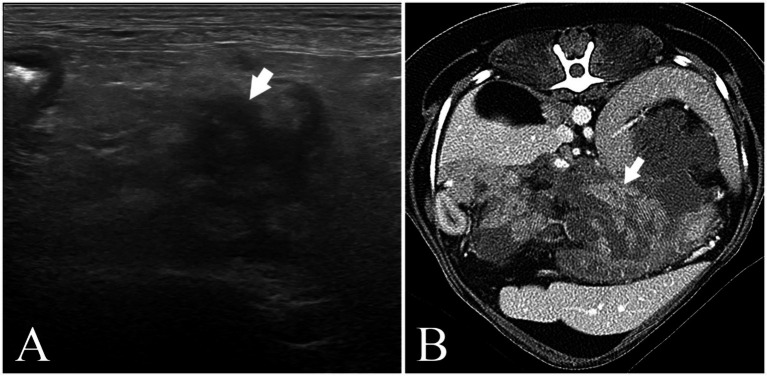
Abdominal ultrasound image (Philips Affiniti 50G) and abdominal CT scan image (Canon Aquilion Lightning) **(A)** (Pomeranian, 9y, CM) abdominal ultrasound image. The pancreas (arrow) is swollen, irregularly marginated, hypoechoic, and the surrounding fat tissue is hyperechoic. His cPL (1,252➔176ng/mL) and CRP (9.2➔1.6 mg/L) decreased after MF-STEM cell treatment. His pancreatitis recurred on the follow-up. **(B)** Abdominal CT scan image. (Bichon Frise, 6y, SF) The pancreas (arrow) is swollen, hypoechoic, and has an irregular margin. His cPL (324➔85ng/mL) and CRP (2.6➔0.1 mg/L) decreased after MF-STEM cell therapy. Furthermore, his bilirubin (1.8➔0.13 mg/L) decreased.

The patients with vomiting and/or diarrhea underwent blood tests, ultrasound tests, CT scans, and other diagnostic tests as needed at the time of admission and a blood test at discharge. Blood samples for the serum chemistry test were collected in a heparin tube. The cPL was measured using commercial ELISA (Vcheck cPL 2.0, Bionote Inc., Hwaseong, Korea) and CRP was evaluated with a serum chemistry diagnostic machine (#Dotto 2000, MTD Medical System, Co.). The cPL and CRP were always measured together. Those two values were measured on the day of hospitalization and every 2 days afterward until the patient was discharged or deceased. Some patients had their cPL and CRP levels evaluated every day if their health was in poor condition or their owners requested. Pre-treatment value was measured on the day of hospitalization, and the post-treatment value was the latest value measured before discharge or death. Alanine aminotransferase (ALT), alkaline phosphatase (ALKP), gamma-glutamyl transferase (GGT), blood urea nitrogen (BUN), creatinine (CREA), and total bilirubin (TBIL) levels were examined at admission to identify concurrent diseases and potentially re-examined later for complications. They were examined later when the patient needed serum chemistry monitoring due to their grave state or concurrent disease. Abdominal ultrasound was performed before all admissions of suspected pancreatitis patients, observing the hyperechoic area due to enlarged pancreas or peripancreatic fat necrosis. The computer tomography (CT) was performed in some patients who needed to rule out pancreatobiliary tumor (Figure 1 and Table 1). The patients were discharged 4 days after admission when they showed no GI signs. If not, therapy continued until they did not show GI signs for 2 days.

**Table 1:** Diagnostic tools and criteria for pancreatitis.

Name		Value	Use
CPL (ng/mL)		≤ 200	Normal
		201–399	Gray zone^1^
		≥400	Diagnosed with pancreatitis
CRP (mg/L)		<1.0	Normal
		≥ 1.0	suspected SIRS
Liver panel	ALT (U/L)	10–109	Liver function test
	ALKP (U/L)	0–214	
	GGT (U/L)	1–14	
Kidney panel	BUN (mg/dL)	8–28	Kidney function test
	CREA (mg/dL)	0.5–1.7	
TBIL (mg/dL)		0–0.9	Biliary function test
Ultrasound		Enlarged and/or hyperechoic pancreas	Axillary diagnostic
CT		Edema and/or effusion	Differential diagnosis with cancer

^1^Severity of pancreatitis was assessed with history, clinical signs, blood exam, and abdominal ultrasound.

ALKP, alkaline phosphatase; ALT, alanine transferase; CPL, canine-specific pancreatic lipase (ng/ml); CRP, c-reactive protein (mg/L); CT, computed tomography; GGT, gamma glutamyl transferase; BUN, blood urea nitrogen; CREA, creatinine; SIRS, systemic inflammatory response syndrome; TBIL, total bilirubin.

Study participants were catheterized and received fluid therapy based on venous blood gas analysis. Patients received antibiotics (metronidazole 15 mg/kg IV or PO BID, METRYNAL INJ., Dai Han Pharm. Co., Ltd.), gastric protectant (famotidine 0.5 mg/kg IV, SC or PO BID, Gaster Inj., Dong-A ST), and antiemetics (maropitant 1 mg/kg SC SID, NOVART Inj., Green Cross Veterinary Products Co., Ltd.) as needed (Table 2). For pain management, buscopan (Buscopan^®^ Compositum 1 mL/kg IV BID, Labiana Life Sciences) or Buprenorphine patch 5ug (Norspan^®^, once every 5 days, LTS Lohmann Therapie-Systeme AG) was administered. The test group was administered the MF-STEM cell (T-STEM PET, 1vial/5 kg IV SID) (Table 3) for various durations (mean 3.09 days, ranging from 1 day to 10 days). Any signs observed following stem cell therapy were documented in detail. Upon discharge, all participants received prescriptions for sulfamethoxazole/trimethoprim (Septrin Tab., 15 mg/kg PO BID, Samil Pharmaceutical Co. Ltd.) and famotidine (Nelson Famotidine Tab, 0.5–1.1 mg/kg PO BID, Nelson Korea Pharm. Co. Ltd.).

**Table 2:** MF-STEM treatment dose by body weight.

0.2 vial/kg, 1 shot per day, 4 shots total	
≤ 5 kg	1 vial/day
5–10 kg*	2 vials/day
10–15 kg**	3 vials/day

*More than 5 kg and includes 10 kg.

**More than 10 kg and includes 15 kg. No patients weighed more than 15 kg.

**Table 3:** Description of gender and respective survival rate by age of study participants.

		Control (*n*)	Test (*n*)
Gender			
Male	Intact	10	3
	Neutered	38	29
Female	Intact	8	10
	Spayed	25	21
Unknown		1	0
Age (years)			
	0–7	15	14
	7–9	9	14
	9–11	9	17
	11–13	26	12
	13+	23	6
Survival (%) by age (years)			
	0–7	40%	100%
	7–9	67%	100%
	9–11	67%	88%
	11–13	46%	92%
	13+	35%	67%

All hospitalized patients were given low fat diet [Intestinal Low Fat (D/L), Dr. Healmedix, or Prescription Diet i/d Low Fat Canine Rice, Vegetable & Chicken Stew, Hill’s Pet Nutrition, Inc]. Inappetent patients were syringe-fed.

In cases of pancreatitis with no suspected SIRS (cPL ≥ 200 ng/mL, CRP < 1.0 mg/L), patient discharge was determined by a decreasing cPL value and the absence of GI signs. Disease progression was defined by an increasing cPL trend was observed. In cases of pancreatitis with suspected SIRS (cPL ≥ 200 ng/mL, CRP ≥ 1.0 mg/L), discharge required confirmation of decreasing cPL and CRP values, along with the absence of GI signs.

### 2.2 Statistical analysis

Experimental values are presented as the mean ± standard deviation based on the average value of the measurement index. Statistical comparisons were performed using the GraphPad PRISM statistical package (version 2.00, GraphPad Software Inc., USA). Significant differences between groups were analyzed using the Student’s paired t-test when the dependent variable was continuous (e.g., cPL and CRP). For categorical dependent variables, the Wilcoxon signed rank test and McNemar test were used for ordinal variables and nominal variables (e.g., survival), respectively. It was considered statistically significant when *p*-value <0.05.

## 3 Results

### 3.1 Descriptive statistics

The gender distribution in the test group was balanced (male = 51%, female 49%), whereas the control group had a higher proportion of males (male = 59%, female = 40%, unknow*n* = 1%) (Table 3). The control group had an overall older distribution compared to the test group. In both groups, more than half of the participants were between 7 and 13 years of age (control: 53%, test: 68%). A general trend of higher survival rates was observed in younger patients (Table 3). The five most common breeds in the study population were Maltese (19%), Pomeranian (14%), Poodle (14%), Mixed (14%), Yorkshire Terrier (12%), and Shih Tzu (11%).

### 3.2 Canine-specific pancreatic lipase (CPL)

**Figure 2:**
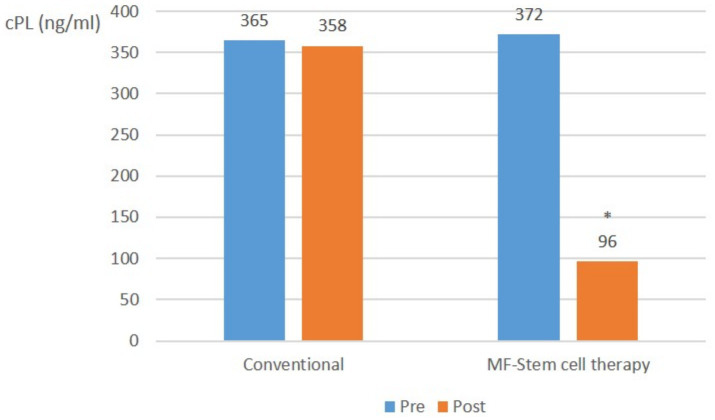
Canine-specific pancreatic lipase (CPL) (ng/ml) average in mild pancreatitis (cPL ≥ 200 ng/mL, CRP < 1.0 mg/L) at pre-treatment and post-treatment (*p < 0.05).

In relatively mild pancreatitis cases, the mean CRP level did not significantly (*p* > 0.05) differ in the control group. However, it significantly (*p* < 0.05) decreased from 372 ng/mL to 96 ng/mL in the test group (Figure 2).

**Figure 3:**
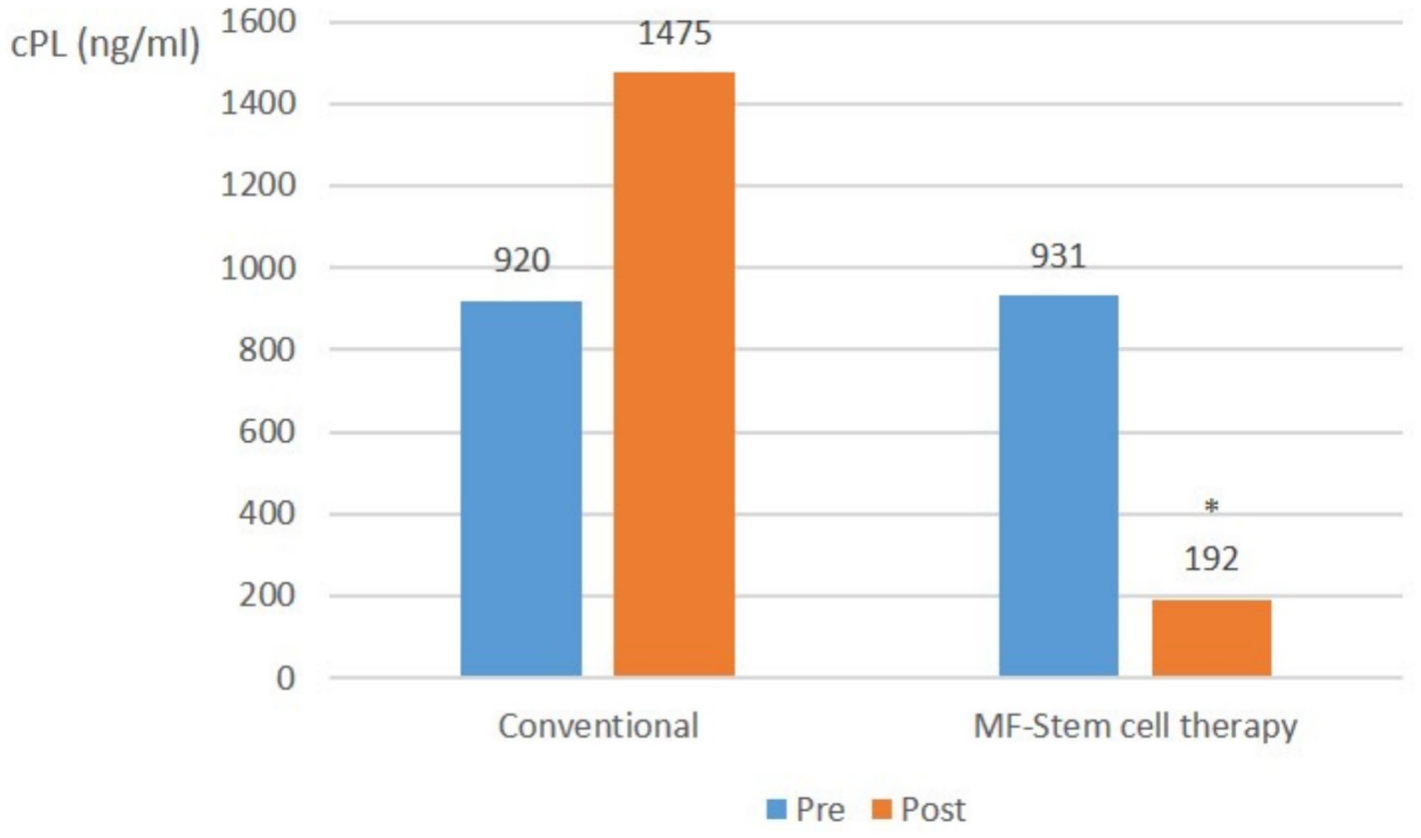
Canine-specific pancreatic lipase (CPL) (ng/ml) changes in severe pancreatitis (cPL ≥ 200 ng/mL, CRP ≥ 1.0 mg/L) with suspected SIRS at pre-treatment and post-treatment (*p < 0.05).

In severe pancreatitis cases (cPL ≥ 200 ng/mL, CRP ≥ 1.0 mg/L), the mean CPL level in the control group significantly (*p* < 0.05) increased from 920 ng/mL to 1,475 ng/mL. Conversely, that of the test group significantly (*p* < 0.05) decreased from 931 ng/mL to 192 ng/mL (Figure 3).

**Figure 4:**
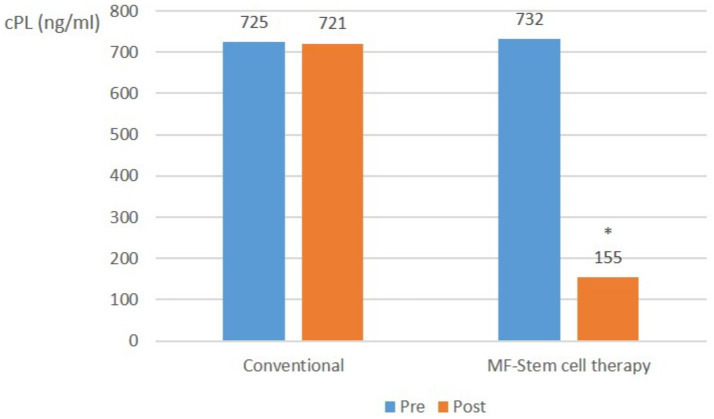
Canine-specific pancreatic lipase (CPL) (ng/ml) changes in all pancreatitis (cPL ≥ 200 ng/mL, CRP ≥ 1.0 mg/L) with suspected SIRS at pre-treatment and post-treatment (*p < 0.05).

Across all pancreatitis cases, there was no significant (*p* > 0.05) change in the average CPL level in the control group. In contrast, the mean cPL level in the test group significantly (*p* < 0.05) decreased from 732 ng/mL to 155 ng/mL (Figure 4).

### 3.3 C-reactive protein (CRP)

**Figure 5:**
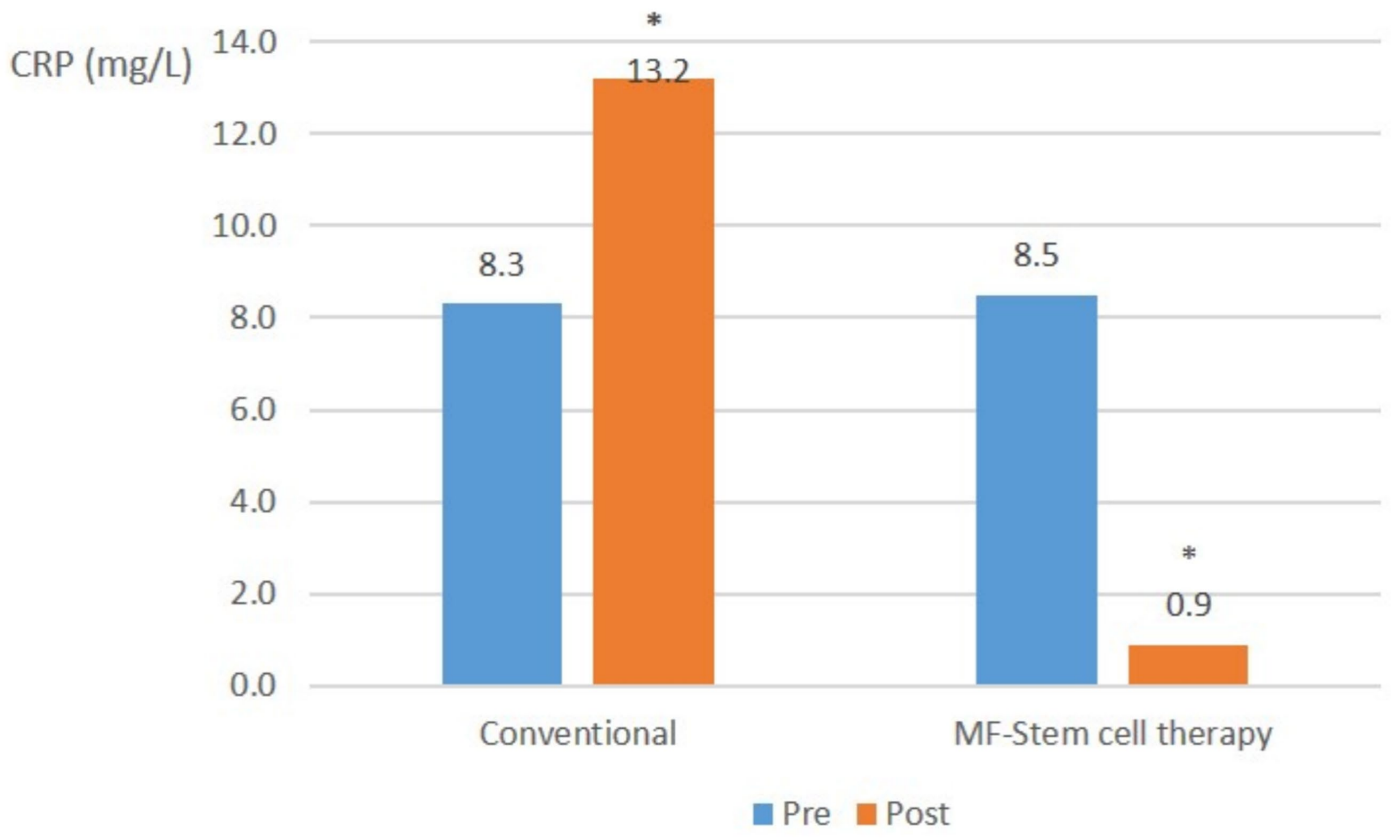
C-reactive protein (CRP) (mg/L) changes in suspected SIRS cases.

In severe pancreatitis cases, the mean CRP level in the control group significantly (*p* < 0.05) increased from 8.3 mg/L to 13.2 mg/L. Conversely, the test group showed a significant (*p* < 0.05) decrease in mean CRP from 8.5 mg/L to 0.9 mg/L (Figure 5).

### 3.4 Survival

**Figure 6:**
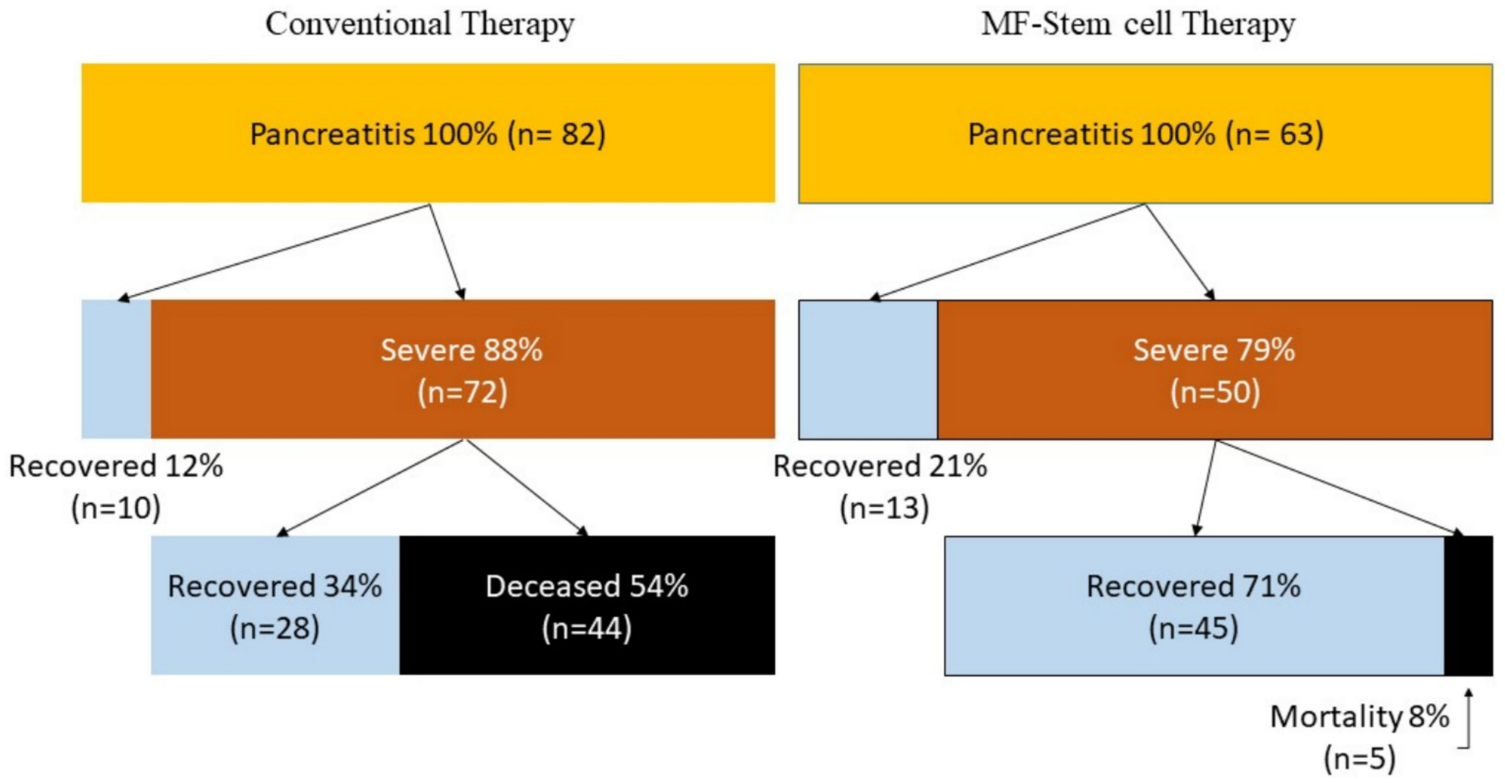
Flow chart of recovery and progression of pancreatitis.

Patients who received MF-stem cell therapy had a survival rate of 92% and a mortality rate of 8%. In contrast, the survival rate was 46% in the control group, with a corresponding mortality rate of 54% (Figure 6).

### 3.5 Blood chemistry test

Some of the liver panels (ALT and ALKP) of the test group showed a significant (*p* < 0.05) decrease compared to the control group. The BUN levels significantly (*p* < 0.05) decreased in both the test group and the control group. However, ALT, ALKP, and BUN remained above the normal range.

### 3.6 Safety evaluation including side effects

No side effects, including allergic reactions, vomiting, and diarrhea, were reported.

### 3.7 Appropriate number of MF-STEM treatments

**Figure 7:**
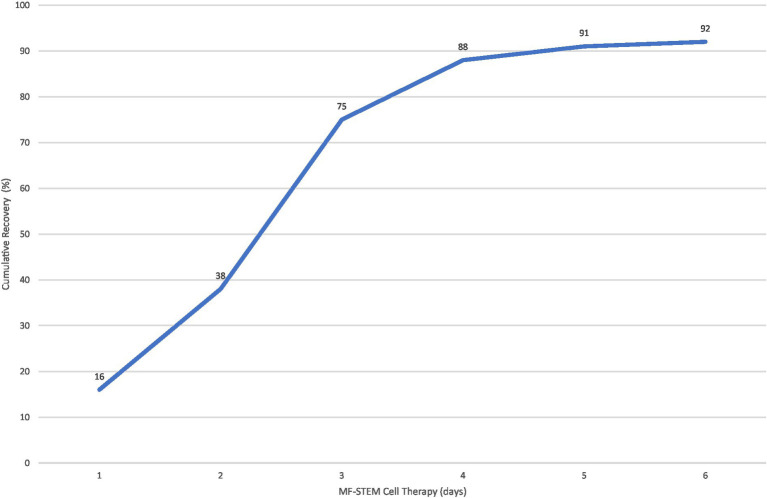
Correlation between treatment number and cumulative percentage (%) of recovered patients.

Eighty-eight percent of dogs survived with 4 or less stem cell treatments (Figure 7).

## 4 Discussion

**Figure 8:**
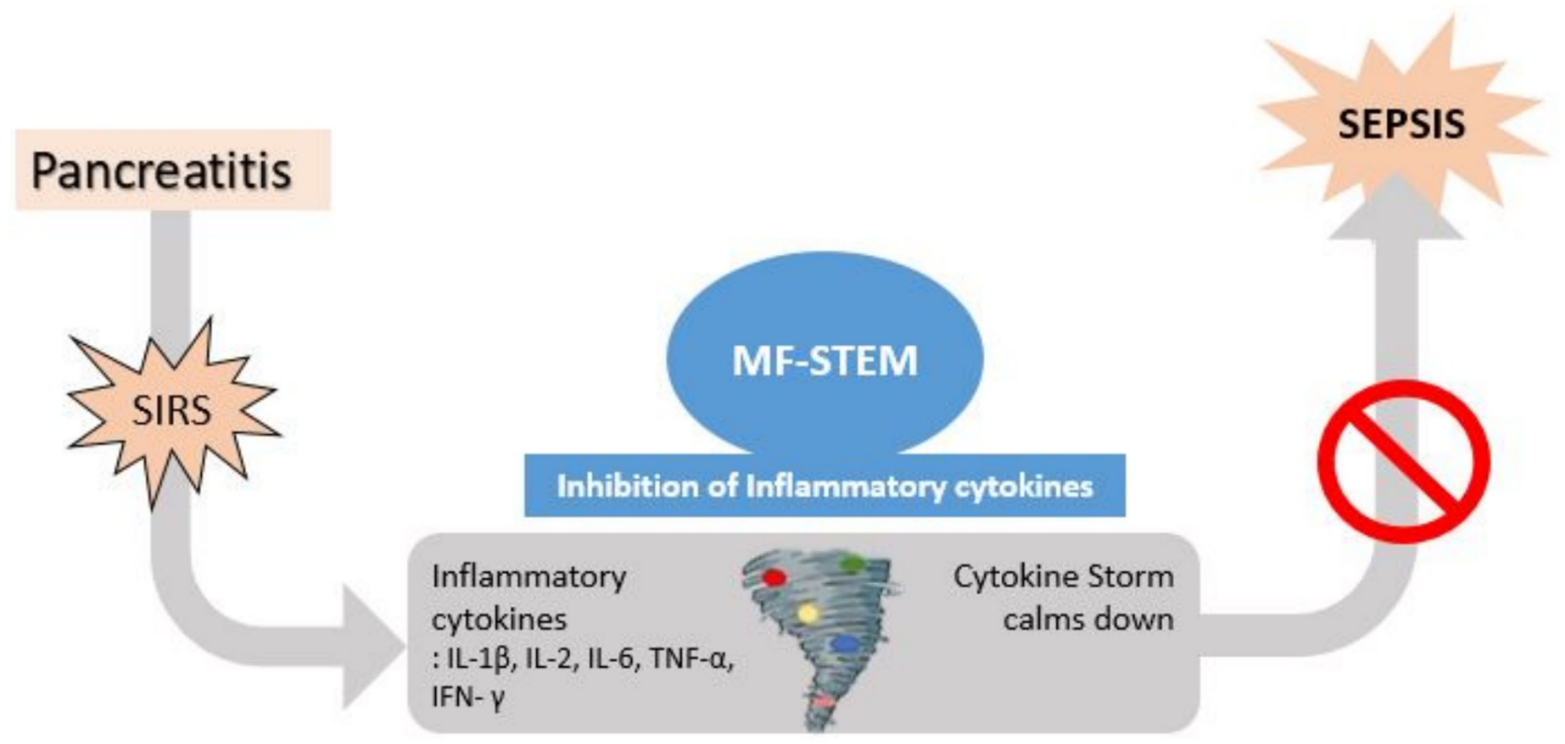
Therapeutic mechanism of MF-STEM.

If pancreatitis progresses without resolution, inflammation can extend beyond the pancreas and progress to SIRS. A cytokine storm can occur in which a variety of inflammatory cytokines, such as IL-1b, IL-2, IL-6, TNF-a, and IFN, are secreted. Previous studies (11, 20) have shown that stem cell therapy can improve histopathological signs (necrosis, edema, hemorrhage, and inflammatory cell infiltration) of the pancreas, decrease inflammatory cytokines (IL-6 and TNF-*α*) and increase anti-inflammatory cytokines (IL-4 and IL-10). Based on these findings, we hypothesized that the MF-STEM may suppress the excessive inflammatory cytokines associated with SIRS, thereby preventing the progression to fatal sepsis (9, 12, 13) (Figure 8).

Canine pancreatitis has numerous potential stressors, including diet, drugs/toxins, concurrent disease, heredity, lipid disorders, and miscellaneous (e.g., age, obesity, and previous surgery) (17). In this study, high-fat diets and prednisolone administration are suspected as the primary causes of pancreatitis in the participants.

*In vitro* analysis revealed that the MF-STEM treatment of macrophages resulted in a concentration-dependent decrease in lipopolysaccharide (LPS)-induced secretion of the inflammatory cytokine TNF-*α*, while the treatment of epithelial cells led to a concentration-dependent increase in the secretion of the regenerative cytokine TGF-ß. MF-STEM contains integrin B1 (ITGB1) and annexin A1 (ANXA1), which have anti-inflammatory effects by inhibiting cytokine release from macrophages. The anti-inflammatory effect of MF-STEM was also demonstrated in LPS-induced RAW264.7 cells (9). Additionally, MF-STEM inhibited interleukin-1α (IL-1α)-stimulated inflammation in primary chondrocytes from mice (13). Both ITGB1 and ANXA1 appear to play a major role in anti-inflammation and regeneration, such as wound healing. The anti-inflammatory effect of ITGB1 was confirmed using LPS-stimulated macrophages (18). The anti-inflammatory and regenerative (wound healing) effects of ANXA1 have also been confirmed in various previous studies (19). Therefore, these findings suggest that MF-STEM possesses both anti-inflammatory and regenerative effects (9, 13).

A complete SIRS profile could not be evaluated in all patients because the complete blood count (CBC) could not be performed on all patients due to financial constraints. However, previous studies (21, 22, 25) support the prognostic value of cPL and CRP in pancreatitis. Furthermore, due to limited resources, participating animal clinics could not provide detailed patient vitality data, which is stored as texts within individual electronic medical records. However, blood test results are available as Excel files upon request.

This study was not double-blinded, raising the possibility that patients in the test group received more frequent attention for side-effect monitoring compared to the control group. However, given the critical condition of all hospitalized participants and the owners’ concern, it is unlikely that patients not receiving MF-STEM were neglected. Additionally, an economic bias may exist, as canine pancreatitis patients who could not be hospitalized were excluded from the study. Finally, the elevated ALT, ALKP, and BUN values likely resulted from the early cessation of serum chemistry monitoring following clinical improvement; however, the observed significant decrease suggests that further monitoring might have revealed values closer to the normal range.

A prior study (3) reported that 36.25% of canine pancreatitis patients also had diabetes mellitus. It is hypothesized that pancreatic *β* acinar cell destruction impairs insulin secretion in dogs (23, 24). Future research will investigate whether the immunomodulatory effects of MF-stem cells can prevent the development of diabetes mellitus in canine pancreatitis patients.

## 5 Conclusion

The MF-STEM treatment has a therapeutic effect, preventing the progression from pancreatitis to SIRS and death. As a final result, it was confirmed that MF-STEM had a significant effect on increasing the survival rate of pancreatitis from 46% (*n* = 38) to 92% (*n* = 58) and reducing the mortality rate from 54% (*n* = 44) to 8% (*n* = 5).

## Data Availability

The original contributions presented in the study are included in the article/supplementary material, further inquiries can be directed to the corresponding author/s.
